# Depression, testosterone concentration, sexual dysfunction and methadone use among men with hypogonadism and HIV infection

**DOI:** 10.1186/1742-4690-9-S1-P141

**Published:** 2012-05-25

**Authors:** Mahmood Amini Lari, Hosain Faramarz, Mesbah Shams, Maryam MarzbanA, Nader Parsa

**Affiliations:** 1 Shiraz Hiv Aids Research Center, Shiraz, Iran

## Background

Hypogonadism is known to be prevalent among patients with HIV infection. Low testosterone levels are associated with depression and impaired sexual performance. The purpose of this study was to determine the impact of hypogonadism on sexual function and depression among men with HIV infection in Shiraz, Iran.

## Material and methods

A total of 237 patients referred to voluntary counseling centers were recruited based on convenience sampling. All patients provided informed consent and completed the Beck Depression Inventory and Brief Male Sexual Function Inventory (BMSFI) from May to October 2009. Early morning fasting blood samples were collected to measure free testosterone (FT) concentration.

## Results

According to FT level, 67.8% of the participants had hypogonadism. About 68% had moderate and severe depression. According to the t-test, serum FT levels were significantly lower in patients with depression (t=1.97 & P=0.046). By univariate regression analysis the odds ratio of having a higher depression score was 1.96 times higher in men with hypogonadism than in eugonadal one (CI: 1.09-3.58). Methadone use was significantly associated with FT (OR = 1.8, 95% CI: 1.01-3.21). We found a significant inverse relationship between sexual drive, erectile and ejaculatory function domains of BMFSI with hypogonadism. In an analysis of four subgroups we investigated the effect of methadone and depression on BMSFI domains. An inverse association was found in methadone non-user and non-depressed patients were seen between hypogonadal and eugonadal men in three domains of BMSFI. However, there was no significant association between methadone user status and depression.

## Conclusion

Depression and hypogonadism had a reciprocal effect. Depression and methadone use were associated with hypogonadism and had significant effects on sexual function.

